# Surgical residents’ career interests in transplantation surgery in Germany – a nationwide survey

**DOI:** 10.3389/ti.2026.15736

**Published:** 2026-07-01

**Authors:** Aghnia Jolanda Putri, Karim Bouhadjer, Arzu Oezcelik, Silvio Nadalin, Markus Guba, Christoph Michalski, Peri Husen

**Affiliations:** 1 Department of General, Visceral, and Transplantation Surgery, University Hospital of Heidelberg, Heidelberg, Germany; 2 Department of General, Visceral, and Transplantation Surgery, University Medicine Essen, Essen, Germany; 3 Department of General, Visceral, and Transplantation Surgery, University Hospital of Tübingen, Tübingen, Germany; 4 Department of General, Visceral, and Transplantation Surgery, LMU University Hospital, Munich, Germany

**Keywords:** medical education, surgical residents, training pathways, transplantation surgery, workforce sustainability

## Abstract

The sustainability of transplantation surgery (TS) relies on the ability to attract and retain the next generation of surgeons. This study aims to assess how surgical residents perceive TS as a career pathway in Germany, and to identify barriers in choosing TS as a career. An anonymous 30-item online survey was distributed to surgical residents at all transplant centers registered with the Deutsche Stiftung Organtransplantation (DSO, German Organ Procurement Organization). Quantitative data were analyzed using SPSS (version 29), free-text responses underwent thematic content analysis. Sixty-eight complete surveys were analyzed. TS was considered by 25% of respondents as a subspecialty training. Transplant-specific education was occasional or absent, though desired by >80%. Motivators included personal interest, patient care, and career development, while barriers were structural: lack of defined fellowship programs and limited autonomy during residency. Structured fellowship programs (46%), mentorship (27%) and enhanced operative autonomy (25%) were identified as key factors to encourage recruitment of surgeons in their early career stages. Surgical residents in their early career stages in Germany regard TS as an appealing career option. A structured training framework is a perceived unmet need to enhance recruitment to TS. Transplantation surgery, workforce sustainability, medical education

## Graphical Abstract

**Figure:**
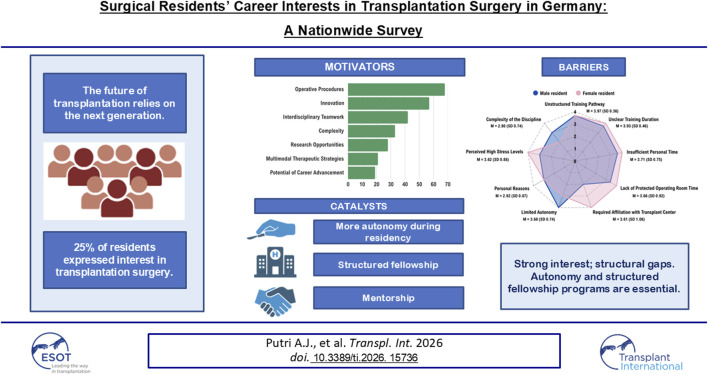

## Introduction

The future of transplantation surgery (TS) depends not only on scientific and technical advances but also on the ability to attract, train, and retain the next-generation of surgeons. Globally, TS faces unique challenges in workforce development due to its complexity, demanding schedules [1, 2], and lack of standardized training pathways in many countries. These challenges are also evident in the German context [3]. Contributing factors include low workforce retention and comparatively modest procedural volumes within the subspecialty [3, 4]. The median caseload per surgeon remains limited, with 16 liver transplants performed annually, and fewer than 20% of these surgeons having completed formal subspecialty training. Loss of early-career surgeons to alternative subspecialties further underscores the systemic difficulties, as most surgeons involved in liver transplantation exit the field after a median of just 3.5 years [4]. This trend potentially compromises the continuity and quality of surgical care.

Beyond structural deficits, gender-specific and generational differences also appear to influence interest and persistence in TS [5]. In Germany, despite increasing numbers of women entering surgical residency, they remain significantly underrepresented in leadership roles and high-complexity subspecialties such as transplantation [6, 7]. To date, however, there has been no systematic national assessment on the sustainability of the transplantation’s workforce, which translates to how surgical residents in Germany perceive TS as a potential career path. Understanding their motivations, barriers, and expectations is critical to informing structural reforms, amid a perceived static or declining interest from trainees.

To assess these perceived challenges and the attitude of young surgical trainees, we conducted a nationwide survey with surgical residents in Germany. Specifically, we sought to (1) evaluate the level of interest in transplantation as a career, (2) identify structural and perceived barriers to pursuing TS, and (3) determine which supportive measures could enhance recruitment and retention. In doing so, we hope to provide a data-driven assessment that allows to form recommendations for strengthening the future transplant surgical workforce in Germany.

## Materials and methods

An anonymous online survey was generated using the LimeSurvey platform^1^. The questionnaire was developed and drafted by two of the authors (AJP, PH). A review of the literature was conducted to identify existing frameworks and validated items that could be adapted to our context. The survey comprised 30 items designed to quantify the prevalence and relative importance of predefined motivators and barriers across a nationwide cohort of surgical residents, and to provide a broad, policy-relevant snapshot of which factors occur most frequently and are perceived as most limiting. To ensure clarity and relevance, the questionnaire was reviewed by experts in the field, including transplant surgeons, educators, and researchers, to validate the questions prior to distribution. The questionnaire was then piloted with 25 medical students rotating in our center. Their feedback, combined with input from experts, helped ensure the instrument’s effectiveness in capturing the intended data and also eliminated any overlap with the targeted resident as samples. The final survey is available in *Supplementary Material 1*. The survey was then disseminated to all university hospitals and transplant centers registered with the *Deutsche Stiftung Organtransplantation* (DSO) across Germany. Contact information were obtained from publicly accessible institutional websites. In cases where individual contact information was unavailable, the survey was distributed via the head of department or the responsible program director. Participants were provided with an informed consent form *prior to* completing the survey, outlining the study’s objectives, voluntary participation, and the use of anonymized data.

Any survey with incomplete responses was excluded prior to analysis to ensure consistency across all reported variables. Quantitative data were analyzed using IBM SPSS Statistics (version 29, IBM Inc., Armonk, New York, USA), with a p-value <0.05 considered statistically significant. The free-text responses are presented as a complementary component to contextualize and illustrate the quantitative findings, which were analyzed using thematic content analysis with iterative coding. All responses were reviewed, coded inductively, and refined into final themes. The study was conducted and reported in accordance with the Checklist for Reporting Results of Internet E-Surveys (CHERRIES) guidelines (see *Supplementary Material 2*). In accordance with institutional guidelines, formal ethics approval was not required for this anonymous, non-interventional survey study involving healthcare professionals. The study was conducted in accordance with the principles of the Declaration of Helsinki.

## Results

### Characteristics of the respondents

A total of 96 responses were received. Out of these, eleven were excluded due to the respondents having already completed their surgical residency training, while another 17 submissions were incomplete and therefore excluded. The mean age of survey participants was 32 years (SD = 0.803), with 93% of respondents being under the age of 35. Of the respondents, 60% (n = 38) identified as female, 40% (n = 29) as male, and one respondent identified as gender neutral. More than half of the respondents (56%) held a German academic title (i.e., *Dr. med.*), and nearly all were employed in full-time clinical positions. The respondents represented all levels of residency training, with the majority being in their fourth year of training. Importantly, 90% of respondents expressed interest in pursuing a subspecialty fellowship in visceral surgery, with 25% of those (*n* = 17) specifically indicating an interest in TS. A higher proportion of men indicated interest in pursuing a TS certification (34.5% of men vs. 18.4% of women), whereas women were slightly more represented among those not interested in any additional certification (13.2% of women vs. 10.3% of men). Among the 68 respondents, 38.2% (*n* = 26) reported having a mentor who is a transplant surgeon. Another 26.5% (*n* = 18) indicated they had a mentor, but not in the field of TS, while 35.3% (*n* = 24) reported having no mentor at all. Regarding the personal characteristics, a significantly higher proportion of male respondents reported being in a committed relationship compared to female respondents (75.9% of men vs. 52.6% of women, p 0.001). Conversely, female residents were more likely to report being single (42.1% of women vs. 24.1% of men, p 0.001). Only 9% of respondents (*n* = 6) reported having children (Table 1).

**TABLE 1:** Characteristic of respondents.

Variables	Sex			All	p
	M (n = 29)	F (n = 38)	Neutral (n = 1)		
Year of residency					0.20
Years 1–3	18 (60)	12 (31)	0 (0)	30	​
Years 4–6	11 (40)	26 (69)	1 (100)	38	​
Academic degree					0.44
No academic degree	13 (45)	19 (50)	0 (0)	32	​
Dr. med	14 (48)	19 (50)	1 (100)	34	​
*Privatdozent*	2 (7)	0 (0)	0	2	​
Employment status					0.44
Full-time	29 (100)	36 (95)	1 (100)	66	​
Part-time (≥75%)	0 (0)	2 (5)	0 (0)	2	​
Interest in pursuing a surgical fellowship					**0.001**
Transplant surgery	10 (35)	7 (18)	1 (100)	18	​
Other surgical fellowship	16 (55)	26 (68)	0 (0)	42	​
Not interested	3 (10)	5 (14)	0 (0)	8	​
Presence of mentor					0.77
Mentored by a Tx surgeon	12 (41)	14 (37)	0 (0)	28	​
Mentored by a non-Tx surgeon	8 (28)	10 (26)	0 (0)	18	​
No mentor	9 (31)	14 (37)	1 (100)	24	​
Relationship status					**0.001**
Single	7 (24)	16 (42)	0 (0)	23	​
Committed	22 (76)	20 (53)	0 (0)	42	​
Prefer not to say	0 (0)	2 (5)	1 (100)	3	​
Parental status					0.45
Parent	4 (14)	2 (5)	0 (0)	6	​
Non-parent	25 (86)	36 (95)	1 (100)	62	​

### Characteristics of the respondents’ training centers

**FIGURE 1:**
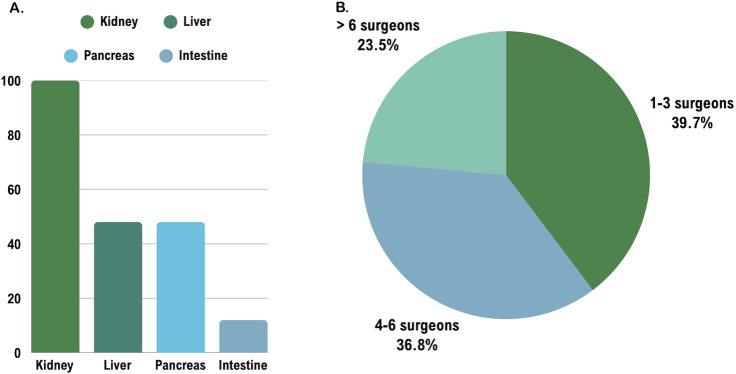
Percentage of **(A)** transplant procedures and **(B)** transplant surgeons at respondents’ training centers.

Respondents were asked to report which types of solid organ transplantation were actively performed at their respective training centers (Figure 1). Kidney transplantation was the most widely available, performed at all participating centers. Liver and pancreas transplantation were each reported as active programs at 71% of respondents’ centers (n = 48), while intestinal transplantation was reported at 12% of respondents’ centers (n = 8). Twenty-seven of respondents reported having 1–3 transplant surgeons in their center, and 25 respondents reported of having 4–6 transplant surgeons. Notably, 23.5% (*n* = 16) of respondents were based at institutions with more than six transplant surgeons. With regard to research involvement, only 23.5% of respondents (n = 16) reported active participation in transplantation-related research activities.

When asked about the availability of transplant-specific continuing education (i.e., seminars and courses) at their training centers, two-thirds of respondents indicated that such opportunities were offered only occasionally. Around one third reported no transplant-related educational activities, like seminar and workshop, at their institution, and only one respondent (1.5%) reported that such training occurred regularly. Despite this limited institutional provision, a strong majority (80.9%, *n* = 55) expressed a desire for more transplant-specific seminars or courses.

### Involvement of surgical residents in transplant surgery

The level of surgical resident involvement varied considerably across different types of transplant procedures. In liver transplantation, half of the respondents (50%, *n* = 34) reported serving as second assistants, while 16.2% (*n* = 11) were involved as first assistants. Only 10.3% (*n* = 7) were not involved at all. Notably, 23.5% (*n* = 16) stated that liver transplantation was not performed at their center. In kidney transplantation, resident involvement was more evenly distributed. The largest proportion (44.1%, *n* = 30) served as first assistants, while 33.8% (*n* = 23) reported serving as second assistants. A smaller subset (5.9% each, *n* = 4) reported either leading the operation under supervision or performing the procedure independently. No involvement was reported by 10% (*n* = 7). In pancreas transplantation, involvement was less pronounced: 48.5% (*n* = 33) reported assisting as second assistants, 27.9% (*n* = 19) as first assistants, and 14.7% (*n* = 10) were not involved. Regarding organ procurement, 45.6% (*n* = 31) had assisted, and 5.9% (*n* = 4) had taken on an active operative role under supervision in the procurement process.

Resident independence in back table or bench preparation was minimal across all organ types. In liver transplantation, 98.5% (*n* = 67) of respondents reported no independence during back table preparation, with only one respondent (1.5%) being able to do the procedure independently. Similarly, for kidney transplantation, only 7.4% (*n* = 5) did the back table work independently. None of the respondents (*n* = 68) had independence in back table preparation for pancreas transplantation.

### Factors influencing the pursuit of a formal fellowship training

**FIGURE 2:**
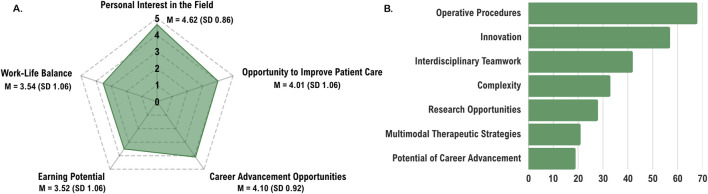
Factors influencing interest in fellowship **(A)** overall **(B)** in transplantation surgery.

Respondents were asked to rate the importance of various factors in their decision to pursue additional subspecialty training using a 5-point Likert scale (1 = not important at all; 5 = very important). The most influential factor was personal interest in the subject matter itself, with a mean score (M) of 4.62 (SD = 0.86), followed by the perceived opportunity to improve patient care (M = 4.01, SD = 1.06) and career advancement prospects (M = 4.10, SD = 0.92). Earning potential (M = 3.53, SD = 1.06) and work-life balance (M = 3.54, SD = 1.06) were rated moderately important (Figure 2A). Further analysis by gender revealed no statistically significant differences between male and female residents regarding the factors influencing their decision to pursue a fellowship.

### Factors influencing the pursuit of transplant surgery as a career option

With regards to which aspects of transplant surgery the respondents found appealing, the operative procedures itself were universally regarded as engaging, with all respondents expressing interest in the surgical aspects of the field (Figure 2B). A substantial majority reported enthusiasm for innovation (83.8%, n = 57), followed by interest in interdisciplinary teamwork (61.8%, n = 42). About 48.5% (n = 33) were drawn to the intellectual and clinical complexity of the specialty while 41.2% (n = 28) expressed interest in the field’s research opportunities. Multimodal therapeutic approaches appealed to 30.9% (n = 21) of respondents. In contrast, only 27.9% (n = 19) viewed career advancement prospects as a motivating factor. No statistically significant gender differences were observed in the distribution of these responses. When asked about their geographic willingness to relocate in order to receive formal training, i.e., fellowship in TS, the majority of respondents (55.9%, n = 38) indicated openness to training opportunities anywhere in the world. An additional 16.2% (n = 11) preferred to remain within Germany, while 11.8% (n = 8) expressed willingness to relocate within the European Union. Only 16.2% (n = 11) reported unwillingness to relocate for a transplant fellowship, mostly due to personal reasons.

Mentorship was identified as a motivating factor by 14.7% (n = 10), who indicated that their interest in TS was inspired by their mentor. Eight respondents (11.8%) reported that they were already interested in TS and intentionally sought out a mentor in the field. A considerable proportion (38.2%, *n* = 26) remained undecided about the mentor’s influence, and 35.3% (*n* = 24) felt that their mentor did not impact their interest in TS.

### Barriers and opportunities: resident perspectives on advancing a career in transplant surgery

**FIGURE 3:**
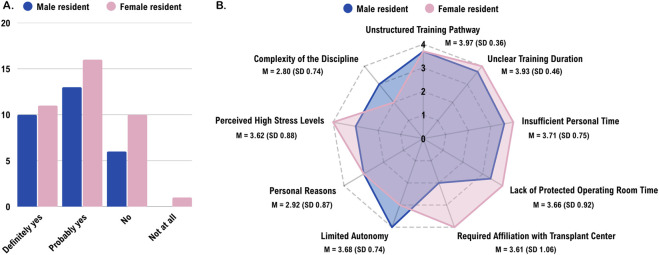
**(A)** Perceived feasibility in having career in transplant surgery **(B)** perceived barriers to residents’ engagement in transplant surgery.

In examining the perceived feasibility to have a career in TS, the majority of respondents expressed a positive inclination (Figure 3A). Specifically, 42.6% (*n* = 29) responded “probably yes” and 30.9% (*n* = 21) selected “definitely yes,” together representing nearly three-quarters (73.5%) of all respondents. Conversely, 23.5% (*n* = 16) indicated “no,” and only a small minority (2.9%, *n* = 2) stated they could not imagine pursuing a career in TS at all. The surgical residents then rated the potential barriers to pursuing a career in TS on a 5-point Likert scale (1 = strongly disagree, 5 = strongly agree) (Figure 3B). The most strongly endorsed barrier was the lack of a structured training pathway (M = 3.97, SD = 0.36) and unclear training duration (M = 3.93, SD = 0.46), followed by a limited operative autonomy during residency (M = 3.68, SD = 0.74). Work-life balance factors also featured prominently, with lack of free time (M = 3.71, SD = 0.75). Additional concerns included the absence of protected operating time for transplant cases (M = 3.66, SD = 0.92) and the perceived constraint of being tied to a transplant center (M = 3.61, SD = 1.06). High stress levels (M = 3.62, SD = 0.88) identified as relevant obstacles. Personal or perception-based factors were viewed as less limiting: private reasons scored lowest (M = 2.92, SD = 0.87), followed by the technical complexity of transplant surgery (M = 2.80, SD = 0.74). The intrinsic challenges of the field itself were not major deterrents for most respondents. No significant gender differences were observed in the perception of these barriers. These findings highlight that structural and systemic barrier, rather than personal preferences or technical concerns, are the predominant deterrents to pursuing a career in transplant surgery.

**FIGURE 4:**
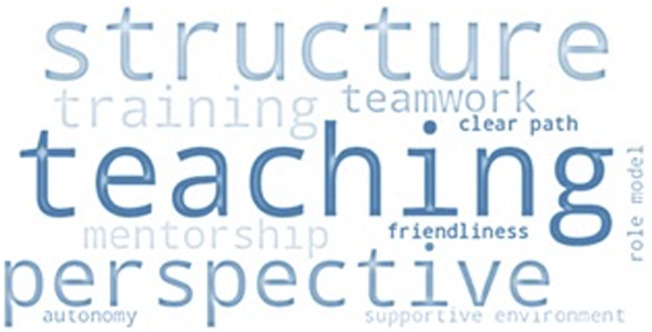
Key drivers of resident engagement in transplant surgery.

Residents predominantly emphasized the need for structured training programs encourage them to pursue a career in TS. Specifically, 45.6% (*n* = 31) selected clear and standardized subspecialty program, such as defined fellowships, as the most critical pulling factor. Mentorship was also highlighted to be important, with 26.5% (*n* = 18) of respondents emphasizing the need for guidance from experienced transplant surgeons. A comparable proportion (25.0%, *n* = 17) underscored the importance of gaining hands-on operative experience during residency, particularly through involvement in smaller procedures such as colorectal surgery or participation in specific steps of hepatopancreatobiliary (HPB) operations. Such exposure was viewed as essential for developing a comprehensive surgical perspective and operative autonomy, thus confident in thinking about career in TS. In contrast, opportunities for research and publication were considered less critical at this stage of training, with only 2.9% (*n* = 2) identifying them as a primary support need. Qualitative responses further emphasized the central role of training structure and autonomy. Several respondents expressed concerns regarding a lack of operative autonomy during training (*n* = 6) and cited this as a deterrent to pursuing the specialty (Figure 4). Other recurring themes included the need for improved working hours and work-life balance (*n* = 5), direct intraoperative teaching (*n* = 5), and greater involvement in perioperative decision-making, including organ allocation decisions (*n* = 5). Further, the absence of engagement from their institutions also serves as a barrier to professional development in TS. Nearly half of the respondents (47.1%, *n* = 32) reported lacking support in pursuing a career in TS from their institutions, while 45.6% (*n* = 31) indicated having partial support. A small minority (7.4%, *n* = 5) felt fully supported in this career path. Notably, a statistically significant association was found between gender and the aspiration to hold a leadership position in transplant surgery (*p* = 0.018). Overall, 75% (n = 51) of respondents expressed a desire to assume leadership roles. When stratified by gender, 89.7% of male respondentsindicated interest in leadership positions, compared to 65.8% of female respondents.

## Discussion

This national survey provides updated insights into the attitudes, exposures, and perceived barriers faced by surgical residents in Germany with respect to having a career in TS from the last decade [3]. Our findings reveal both significant latent interest in the field and notable structural and educational shortcomings that hinder the translation of this interest into formal career progression. These results mirror, and in some aspects update, findings from previous workforce assessments among transplant surgeons and trainees in Germany and across Europe [8, 9]. The data increasingly suggest that Germany is facing a pipeline crisis. A national survey among over 1,000 German medical students revealed that only 14% planned to enter a surgical specialty of any kind. Among female students, this number drops even further to just 11%, which is especially notable considering they comprise 64% of the student population [10]. The translation from surgical residency to a transplant surgeon represents the next critical hurdle [4]. Importantly, this concern is not confined to Germany. Both the United States and the United Kingdom, despite having more structured surgical training pathways, are facing mounting challenges in sustaining a robust transplant workforce [8, 11, 12].

Nearly three-quarters of respondents expressed a willingness to consider a career in TS. This level of expressed interest is encouraging and suggests that TS continues to exert a meaningful appeal among trainees. Operative procedures, innovative techniques, and interdisciplinary teamwork were ranked among the most appealing aspects of the specialty, consistent with prior research on motivational factors in both general and TS. However, the observed enthusiasm sharply contrasts the actual structural environment in which residents are trained. Exposure to transplant procedures remains highly dependent on institutional setting and mentorship [2]. Operative autonomy during residency remains a critical issue in surgeon’s attrition, not limited to transplant procedures alone [3, 13, 14]. Back table preparation, an essential component of the transplant surgical process and a proxy for procedural trust and responsibility, was seldom performed independently. Only one respondent reported independent performance for liver grafts, and none for kidney and pancreas. This echoes previous concerns regarding limited hands-on exposure among trainees and reflects a training environment that continues to prioritize technical performance by senior staff, often at the expense of skill transfer. Interest, and thus, attrition in TS was associated with lower operative volumes, a narrower case mix, and worse patient and graft survival after liver transplantation [2, 9, 15].

To enhance early exposure during residency, it would be beneficial to partially integrate a transplant surgery curriculum. While this is challenging due to the limited number of transplant centers and the constraints of the existing Facharzt training structure in Germany, a structured transplant rotation for those training at the DSO-registered transplant centers should be encouraged, similar to models in the United States. Such rotations would standardize baseline exposure across institutions and ensure all surgical residents acquire essential competencies in transplant surgery, including perioperative management, pre-transplant evaluation, waiting-list processes, complication management (e.g., hyponatremia, spontaneous bacterial peritonitis, refractory ascites), and participation in multidisciplinary kidney and liver selection committee meetings. Programs should promote progressive operative autonomy through staged responsibility models to enhance motivation, skill development, and retention. Additionally, virtual simulation tools and targeted workshops could enhance exposure to transplant scenarios, including operative procedures and management related to the waiting list. These solutions would be particularly beneficial in centers with lower case volumes, aiming to increase hands-on experience and decision-making autonomy for residents.

It has been shown that an effective transplant surgery fellowship should offer substantial case volume, high procedural complexity, and operative autonomy, enabling trainees to independently manage both surgical and nonsurgical aspects of transplant patient care. This educational triad has been repeatedly emphasized as essential for skill maturation and professional identity formation in surgical subspecialties such as transplant and HPB surgery [11, 14]. These concerns are corroborated by prior national studies showing that only a minority of German transplant surgeons dedicate more than 10% of their operative time to transplantation, with most maintaining broader clinical identities in HPB or general surgery [2, 3, 6].

A potential framework for a transplant fellowship could involve a structured, stepwise educational curriculum. The initial 6 months could focus on donor procedures, including multi-visceral deceased donor organ procurement and first-assist roles in transplant cases. This foundational phase would be complemented by research projects, journal clubs, and literature review sessions led by senior staff members, aimed at providing in-depth preparation for the subsequent rotations. These rotations would include living donor procedures, as well as kidney, pancreas, and liver transplants, with fellows ultimately performing these procedures as the lead surgeon. To ensure comprehensive skill development and exposure, the fellowship training should be completed over two consecutive years. Fellows would progress from “junior” status in the first year to “senior” status in the second year, providing a clear structure for training progression. A two-year timeframe would establish a practical and focused training period, addressing the current lack of a defined fellowship pathway in Germany, where training is often unstructured and lacks consistency across institutions. Furthermore, identifying “certified” training centers, typically the largest and most experienced institutions in the country, would ensure that fellows receive maximum exposure to high-volume cases within this structured period. In France, the cardiovascular surgical residency program exemplified how early, dedicated exposure combined with structured mentorship can effectively promote operative autonomy and interest among residents, including in TS [16]. Here, the DSO could also play a role in establishing a national minimum framework for transplant training by defining standardized exposure requirements for trainees at accredited centers. In addition, the DSO is well positioned to develop and facilitate transplant-related educational courses, such as those focused on organ donation processes and the associated logistical infrastructure, to further support uniform, high-quality training across institutions.

Work-life balance and emotional workload were also noted as barriers, although they appeared to be secondary to the structural constraints. This is an important distinction. While lifestyle factors have traditionally been assumed to deter younger physicians from demanding specialties such as transplantation [2, 17, 18], our findings suggest that these factors play a comparatively smaller role. The interest is clearly present, yet a discrepancy persists, including academically [18]. While 41.2% of respondents indicated an interest in research opportunities within TS, only 23.5% were actively involved in transplantation-related research. This mismatch is not unique to transplant surgery and reflects broader trends in surgical training. It is important to note that protected research time and mentoring consistently shown to yield significant long-term academic benefits. It is strongly associated with higher publication productivity, a greater likelihood of obtaining external funding, an increased probability of pursuing a career in academic surgery, and improved progression into leadership roles [19].

Our study identifies a significant gender gap in leadership aspirations (89.7 percent among male residents versus 65.8 percent among female residents), consistent with findings from previous studies. Addressing this disparity requires structured mentorship and active sponsorship, transparent leadership pathways, systematic monitoring of operative autonomy, early leadership development during residency, family-compatible training structures without career penalty, and greater visibility of female role models in transplantation surgery.

Mentorship, widely recognized as a key driver of career development, was inconsistently available as per our analysis. In contrast, in systems with well-established fellowship structures, such as the ASTS model in the United States, mentorship has been shown to play a formative role in guiding young surgeons toward transplant careers [1, 2, 18]. Our data suggest that while mentorship does occur, it is neither systematic nor sufficient to guide career formation in transplantation in the current German context.

A critical aspect, and a limitation of this survey, is the fact that it is aimed at residents only. While the overall interest and motivation to pursue a career in TS is there, a survey to follow up on the development of this motivation at a later age would be equally important in order to see how perceptions develop following graduation from a residency program, which will translate to more operative autonomy after becoming a surgeon (*Facharzt/Fachärztin*). A wide range of trainees at institutions without a transplant program were not addressed here, even though their interest in pursuing a career in transplant surgery or their interest in exposure or rotations to transplant programs would be of interest.

## Conclusion

In summary, this study highlights a critical tension between the enduring appeal of TS to surgical residents in Germany and the structural improvement options of the current training environment. When surgical training does not consistently translate into long-term commitment to subspecialties like TS, the field faces a serious challenge. Transplant surgery, by its nature, demands high levels of skill, dedication, and institutional support. Yet without structural improvements, its future workforce may remain vulnerable. Systematic educational reforms during residency, implementation of structured fellowship programs, and improved mentorship are possible ways to enhance recruitment to transplant surgery and positively influence workforce sustainability.

## Data Availability

The raw data supporting the conclusions of this article will be made available by the authors, without undue reservation.

## Footnotes

1
www.limesurvey.org
