# Supplementary material for: Surgical residents’ career interests in transplantation surgery in Germany – a nationwide survey
Source: Transpl Int. 2026 Jul 1;39:15736. doi: 10.3389/ti.2026.15736 (PMC13368692; doi:10.3389/ti.2026.15736)
Supplement: Supplementary file 1 [file DataSheet2.pdf]

Supplementary Data 2
Table 2. CHERRIES Checklist for Reporting Results of Internet E-Surveys

**Item Category**	**Checklist Item**	**Response**
Design	Target population and sample frame	Surgical residents training at DSO-registered transplant centers in Germany.
Design	Survey design	Cross-sectional anonymous online survey consisting of 30 items assessing career interest, barriers, and supportive measures in transplantation surgery.
IRB & Consent	IRB approval	Formal ethics approval was not required for this anonymous, non-interventional survey according to institutional guidelines. Study conducted in accordance with the Declaration of Helsinki.
IRB & Consent	Informed consent	Participants were provided an electronic consent statement before accessing the questionnaire outlining study purpose, voluntary participation, and anonymous data use.
IRB & Consent	Data protection	No identifying information collected; responses stored anonymously within the LimeSurvey platform.
Development	Questionnaire development	Survey items were developed after literature review and reviewed by transplant surgeons, educators, and researchers for content validity.
Development	Pre-testing	Pilot testing was conducted with 25 medical students rotating at the authors’ institution; feedback improved clarity and removed overlapping items.
Recruitment	Survey type	Closed survey distributed to transplant centers registered with the DSO.

Recruitment	Contact mode	Survey link distributed via email through transplant departments and program directors.
Recruitment	Advertising	No public advertising; distribution limited to institutional contacts.
Administration	Survey platform	Web-based survey administered using LimeSurvey with automatic electronic data capture.
Administration	Participation	Participation was voluntary; no incentives were offered.
Administration	Questionnaire structure	30 items presented across multiple pages in the LimeSurvey interface.
Administration	Completeness check	Incomplete questionnaires were excluded from analysis.
Administration	Review option	Participants could review and modify responses before submission.
Response rates	Participation and completion	96 responses received; 68 complete surveys included in the final analysis.
Duplicate prevention	Multiple entries	No cookies or IP tracking were used in order to maintain anonymity.
Analysis	Handling incomplete questionnaires	Only complete questionnaires were analyzed.
Analysis	Statistical analysis	Quantitative analysis performed using IBM SPSS Statistics version 29; exploratory comparisons considered significant at $p < 0.05$.
