# Supplementary material for: Surgical residents’ career interests in transplantation surgery in Germany – a nationwide survey
Source: Transpl Int. 2026 Jul 1;39:15736. doi: 10.3389/ti.2026.15736 (PMC13368692; doi:10.3389/ti.2026.15736)
Supplement: Supplementary file 2 [file DataSheet1.pdf]

Umfrage Transplantationschirurgie

Umfrage: Zukunftsperspektiven in der Chirurgie – Interesse an der Transplantationschirurgie

Liebe Kolleginnen und Kollegen,
im Rahmen der kontinuierlichen Weiterentwicklung der chirurgischen Weiterbildung möchten wir mehr über Ihre beruflichen Interessen und Ihre Vorstellungen zur Spezialisierung in der Chirurgie erfahren. Die Transplantationschirurgie stellt eine anspruchsvolle und besonders vielseitige Spezialisierung dar. Wir sind daran interessiert zu erfahren, wie Sie zu diesem Fachgebiet als Karriereoption stehen.
Bitte nehmen Sie sich einen Moment Zeit, um diese kurze Umfrage zu beantworten (Dauer ca. 5min).

Demographische Daten

1. **Wie alt sind Sie?**
   - 20-25
   - 26-30
   - 31-35
   - 36-40
   - > 40

2. **Geschlecht**
   - Männlich
   - Weiblich
   - Keine Angabe

3. **Partnerschaft**
   - Ich lebe in einer festen Beziehung
   - Ich bin Single
   - Keine Angabe

4. **Haben Sie Kinder?**
   - Ja
   - Nein

Chirurgische / wissenschaftliche Erfahrung

5. **Welches ist Ihr höchster bereits erworbener akademischer Titel?**
   - Ich habe noch keinen
   - Dr. med.
   - PD Dr. med.
   - Sonstiges: ____________ (Freitext)

6. **Wie ist Ihr aktueller Beschäftigungsstatus?**
   - Vollzeit
   - Teilzeit (≥75 %)
   - Teilzeit (<75 %)
   - Derzeit nicht klinisch tätig (z.B. wegen Forschung, Elternzeit)

7. **Sind Sie Fachärztin/Facharzt für ein chirurgisches Fach?**

   b. Ja, für das Fach Allgemeinchirurgie
   c. Ja, für das Fach Viszeralchirurgie
   d. Ja, für ein anderes chirurgisches Fach
   e. Nein, ich befinde mich noch in der Weiterbildung

8. **In welchem Jahr der Weiterbildung befinden Sie sich?**
   - 1
   - 2

- 3
- 4
- 5
- 6
- Facharztweiterbildung bereits abgeschlossen

9. **Streben Sie den Erwerb einer Zusatzbezeichnung an (z.B. spezielle Viszeralchirurgie, Transplantationsmedizin)?**
    - Ja, für Transplantationsmedizin
    - Ja, für spezielle Viszeralchirurgie
    - Ja, für ein anderes Fachgebiet (bitte unter „Kommentar“ eintragen)
    - Nein

10. **Welche Faktoren sind Ihnen für die Wahl einer Zusatzbezeichnung wichtig? (Skala 1-5, wobei 1 = sehr unwichtig und 5 = sehr wichtig)**
    - Eigenes Interesse am Thema
    - Beitrag zur Patientenversorgung
    - Karrierechancen
    - Verdienstmöglichkeiten
    - Lebensqualität und Work-Life-Balance

Interesse Transplantationschirurgie

11. **In meiner Klinik finden Transplantationen der folgenden Organe statt (Mehrfachnennung möglich):**
    - Leber
    - Niere
    - Pankreas
    - Dünndarm

12. **Wie würden Sie Ihr Interesse an der Transplantationschirurgie einschätzen?** *(Likert-Skala)*
    - Überhaupt nicht interessiert
    - Wenig interessiert
    - Neutral
    - Interessiert
    - Sehr interessiert

13. **Könnten Sie sich vorstellen, zukünftig und langfristig in der Transplantationschirurgie zu arbeiten?**
    - Ja, auf jeden Fall
    - Ja, vielleicht
    - Nein, eher nicht
    - Nein, auf keinen Fall
    - Ich bin noch unentschlossen

14. **Welche Aspekte der Transplantationschirurgie interessieren Sie am meisten? (Mehrfachnennungen möglich)**
    - Organtransplantationen an sich (Operation)
    - Multimodale Therapieansätze (z.B. prä- und post-operative Betreuung)
    - Chirurgische Techniken und Innovationen
    - Die Herausforderung, schwerkranke Patienten zu behandeln
    - Zusammenarbeit im interdisziplinären Team
    - Forschung und klinische Studien
    - Karrierechancen

15. **Sind Sie wissenschaftlich im Bereich der Transplantationsmedizin tätig?**
   - Ja
   - Nein, in einem anderen Gebiet
   - Nein

16. **An meiner Klinik gibt es interne Weiterbildungsveranstaltungen für Assistenten/-innen zum Thema Transplantation:**
   - Ja, regelmäßig
   - Ja, gelegentlich
   - Nein, überhaupt nicht

17. **Wünschen Sie sich mehr interne Fortbildungs-Veranstaltungen zum Thema Transplantationsmedizin/-chirurgie (z.B. Journal Club)**
   - Ja
   - Nein

18. **Wie viele Chirurginnen/Chirurgen transplantieren in Ihrer Klinik selbständig?**
   - 1-3
   - 4-6
   - >6

19. **Wenn eine <u>Leber</u>transplantation stattfindet, bin ich in folgender Weise an der Operation beteiligt:**
   - 2. Assistent/-in
   - 1. Assistent/-in
   - Operateur/-in
   - Gar nicht, werde nicht involviert
   - Findet an unserem Zentrum nicht statt

20. **Wenn eine <u>Nieren</u>transplantation stattfindet, bin ich in folgender Weise an der Operation beteiligt:**
   - 2. Assistent/-in
   - 1. Assistent/-in
   - Operateur/-in
   - Gar nicht
   - Findet an unserem Zentrum nicht statt

21. **Wenn eine <u>Pankreas</u>transplantation stattfindet, bin ich in folgender Weise an der Operation beteiligt:**
   - 2. Assistent/-in
   - 1. Assistent/-in
   - Operateur/-in
   - Gar nicht
   - Findet an unserem Zentrum nicht statt

22. **Ich nehme an Organentnahmen (DSO) bei hirntoten Spendern teil:**
   - Ja, als verantwortliche/-r Entnahmechirurg/-in
   - Ja, als 1. Assistent/-in
   - Nein, bin nicht involviert
   - Nein, unser Zentrum hat kein Entnahmeteam

23. **Beherrschen Sie die Bench / Back Table Präparation für folgende Organe vor der Implantation <u>selbständig?</u> (Mehrfachnennung möglich):**
   - Leber
   - Niere

- Pankreas
- Keine

24. **Haben Sie einen Mentor/eine Mentorin, der/die selbständig Transplantationen durchführt?**
    - Ja
    - Nein, mein Mentor/meine Mentorin ist in einem anderen Gebiet tätig
    - Ich habe keinen Mentor / keine Mentorin

25. **Inwiefern hat Ihr Mentor / Ihre Mentorin Ihre Einstellung zur Transplantationschirurgie beeinflusst?** *(Mehrfachauswahl möglich)*
    - Ich habe Interesse an der Transplantationschirurgie *wegen* meines Mentors / meiner Mentorin entwickelt
    - Ich hatte bereits Interesse an der Transplantationschirurgie und habe mir deshalb gezielt einen Mentor / eine Mentorin aus diesem Bereich gesucht
    - Mein Mentor / meine Mentorin hat mein Interesse an der Transplantationschirurgie eher nicht beeinflusst
    - Ich bin noch unentschlossen
    - Sonstiges

26. **Inwieweit schränken die folgenden Faktoren Ihrer Meinung nach Ihr persönliches Engagement in der Transplantationschirurgie ein?**
    (Likert Skala: Stimme überhaupt nicht zu, Stimme nicht zu, Neutral, Stimme zu, Stimme voll und ganz zu)
    - Unstrukturierte Weiterbildung
    - Unklare Weiterbildungsdauer
    - Wenig Autonomie nach Abschluss der Weiterbildung
    - Hohe psychische Belastung und Stress
    - Mangel an Freizeit
    - Komplexität der Fälle, lange Operationen
    - Keine geregelten Arbeitszeiten, Operieren nachts und an Wochenenden
    - Bindung an ein Transplantationszentrum, wenig Flexibilität
    - Private Gründe (z.B. Familie)

27. **Welche Unterstützung würden Sie benötigen, um sich für eine Spezialisierung in der Transplantationschirurgie zu entscheiden?**
    - Praktische Erfahrungen während der Weiterbildung
    - Mentoring durch erfahrene Transplantationschirurgen/-innen
    - Fort- und Weiterbildungsprogramme mit klarer Struktur und zeitlichem Rahmen (z.B. 2 Jahre Fellowship nach der Facharztprüfung)
    - Forschungs- und Publikationsmöglichkeiten
    - Andere: _______________

28. **Wären Sie bereit, Ihren Wohnort für die Zeit der strukturierten Zusatzweiterbildung in einem großen Transplantationszentrum (z.B. 2 Jahre Fellowship) zu verlegen:**
    - Ja, innerhalb Deutschlands
    - Ja, innerhalb Europas
    - Ja, auch außerhalb Europas
    - Nein, weil______(Freitext)

29. **Streben Sie eine Führungsposition in der Chirurgie an (z. B. als Klinikdirektor/-in oder leitende/-r Oberarzt/-ärztin)?**

- Ja
- Nein
- Nein, weil (Freitext)

30. Fühlen Sie sich von Ihrer Klinik ausreichend unterstützt, was eine mögliche Karriere in der Transplantationschirurgie betrifft?

- Ja
- Teilweise
- Nein
- Nein, weil (Freitext)

31. **Was sollte Ihrer Ansicht nach grundsätzlich verbessert werden, um die Transplantationschirurgie für Assistentinnen/Assistenten interessanter zu machen?**

Freitext

Vielen Dank für Ihre Teilnahme an dieser Umfrage!
Ihre Antworten helfen uns, die Bedürfnisse und Interessen in der chirurgischen Weiterbildung besser zu verstehen und zukünftige Programme entsprechend anzupassen.
